# Clinical and Radiographic Evaluation of a Novel Triangular Implant Neck Design: A Case Series

**DOI:** 10.3390/dj10060113

**Published:** 2022-06-16

**Authors:** James Rudolph Collins, Brendha P. Ogando, Houlin Hong, Wei Hou, Georgios E. Romanos

**Affiliations:** 1 Department of Periodontology, School of Dentistry, Pontificia Universidad Católica Madre y Maestra (PUCMM), Santo Domingo 10109, Dominican Republic; jamescollins@pucmm.edu.do (J.R.C.); brendhaogando@gmail.com (B.P.O.); 2 School of Public Health & Health Policy, City University of New York, New York, NY 10031, USA; hong.houlin@gmail.com; 3 Department of Family, Population and Preventive Medicine, School of Medicine, Stony Brook University, New York, NY 11794, USA; wei.hou@stonybrookmedicine.edu; 4 Department of Periodontology, School of Dental Medicine, Stony Brook University, New York, NY 11794, USA

**Keywords:** dental implants, clinical study, marginal bone loss, implant design

## Abstract

The objective of this study was to evaluate the clinical and radiographic behavior of a novel triangular neck implant configuration in partially edentulous patients. Sixteen patients with a mean age of 58.3 years, were rehabilitated with 25 implants inserted in the healed sites of the maxilla and mandible; implant diameter was Ø3.3 and 3.9 mm. Clinical and radiographic measurements were first performed at prosthesis delivery that served as baseline; they were further evaluated after a mean period of 15.6 months. The interproximal peri-implant bone levels were the primary outcome; the mesial and distal data were recorded and a mean value was calculated. Secondary outcomes included peri-implant probing depth (PPD) and bleeding on probing (BoP). The paired *t*-test was used to compare the radiographic and clinical outcomes between baseline and follow-up. The mean bone levels at the mesial and distal aspects at baseline were 0.45 (0.47) and 0.57 (0.69), respectively; at follow-up they were 0.59 (0.42) and 0.78 (0.59), respectively. The differences were not statistically significant. Similarly, no significant differences were found for the clinical parameters. Within the limitations of the present study, it could be concluded that this new triangular neck bone level implant macro-design was used successfully to treat partially edentulous patients. Larger controlled clinical studies are warranted to confirm the present radiographic and clinical findings.

## 1. Introduction

Over recent decades, development of standardized protocols, technological innovations and new knowledge of the biological processes that occur around dental implants has contributed significantly to the development of oral implantology and dentistry [1]. Osseointegration was originally described as the direct structural and functional connection between living bone and the surface of the loaded implant, which can be assessed microscopically [2]. The main objective of dental implants is to restore masticatory function and aesthetics, thus improving the quality of life of partially and fully edentulous patients [3,4]. Currently, dental implant procedures are becoming more predictable, allowing patients to obtain simpler and less invasive treatments, with long-term clinically successful restorations [5,6,7,8].

The primary and secondary stability of the implant is determined by the features of the micro- and macro-design [9]. Various investigations have shown that the micro-design features can stimulate the migration, growth and adhesion of cells, proteins and growth factors to the implant surface [8,10,11,12]. The macro-design of an implant mainly refers to body and neck shape, thread geometry and pitch distances [13]. Regarding the implant neck design, it has been shown that straight or convergent neck design cause least stress at insertion compared to the divergent or wider neck shape [14,15].

New implant designs and protocols are constantly introduced by dental implant manufacturers, and some of them are positively impacting the reliability of implant therapy [16,17,18]. Moreover, proper diagnostic case selection, management of hard and soft tissues at the time of surgery, platform-switching or conical implant-abutment connections, three-dimensional implant position at the time of placement, and correct prosthetic management are essential for the success of the final implant restorations [19,20,21,22,23,24,25,26].

Recently, an implant with a triangular neck configuration became available on the market [27,28,29]. Several clinical studies have demonstrated that this triangle neck design can achieve primary stability, proper osseointegration, stability of the peri-implant tissues and patient satisfaction [28,29,30]. Li Manni et al. [29] conducted a randomized controlled trial to compare the peri-implant bone changes at a circular and triangular shaped neck implant designs. After 1 year of loading, the proximal bone loss was 0.22 ± 0.30 mm for the triangular neck and 0.42 ± 0.67 mm for the circular ones (*p* = 0.25). In another study by Eshkol-Yogev et al. [28] involving the same neck design, early changes in implant stability were evaluated in the posterior maxilla. The initial ISQ value of the tested triangular neck implant was enough to allow immediate loading (mean 68.4, SD = 8.4) but the mean initial ISQ of the round neck implants (control) was significantly higher at implant placement (mean 76.9, SD = 8.7); these differences, however, disappeared after 6 weeks of healing. In a retrospective study, clinical and radiographic outcomes were evaluated in the esthetic zone after one-year of receiving the triangular neck implant design. The results showed the preservation of hard and soft tissue using this implant design in the esthetic anterior maxilla after one year of function [31]. In another study involving human bio-spies, Nevins et al. [32] showed that the 0.2 mm gap between the implant surface and the buccal cortical bone created in the flat portion of the triangle was filled with bone after 6 months of submerged healing.

The primary aim of the present study was to evaluate the radiographic bone changes around a novel triangular neck bone level implant design in partially edentulous patients after the final restoration was placed. Secondary aims were to verify various clinical parameters, such as, peri-implant probing depth (PPD) and bleeding on probing (BoP).

## 2. Materials and Methods

### 2.1. Patient Selection

This study was conducted in the Department of Periodontics of the School of Dentistry of the Pontificia Universidad Católica Madre y Maestra (PUCMM), Campus Santo Domingo. This protocol was compliant with the Helsinki Declaration of 1975, as revised in 2000. The Ethical Committee of the Faculty of Health approved the protocol (COBE-FACS-M.EST-CSTA-002-2-2015-2016). The protocol was explained to all participants and, after answering every question, they signed the informed consent prior to any treatment. Sixteen patients (4 male and 12 female), with a mean age at placement of 45.82 (±13.5) years, requiring one or more implant restorations were treated between July 2016 and November 2019. Inclusion criteria were, as follows: adults ≥ 18 years of age; one or more missing teeth in the anterior or posterior maxilla or mandible; availability for the duration of the study; signing the informed consent form; good general health and oral hygiene (O’Leary plaque score of ≤20%). Exclusion criteria included: presence of oral pathology, untreated active periodontal disease, patients with total upper and lower edentulism, need for regenerative procedures at the implant site, pregnant or breastfeeding women and smokers.

All subjects underwent an initial clinical and radiographic evaluation that included CBCT images, for diagnostic and treatment planning. After verifying the inclusion and exclusion criteria of the study, an experienced investigator (JC) screened, selected, and performed all implant surgeries for the participants.

### 2.2. Surgical Procedures

**Figure 1:**
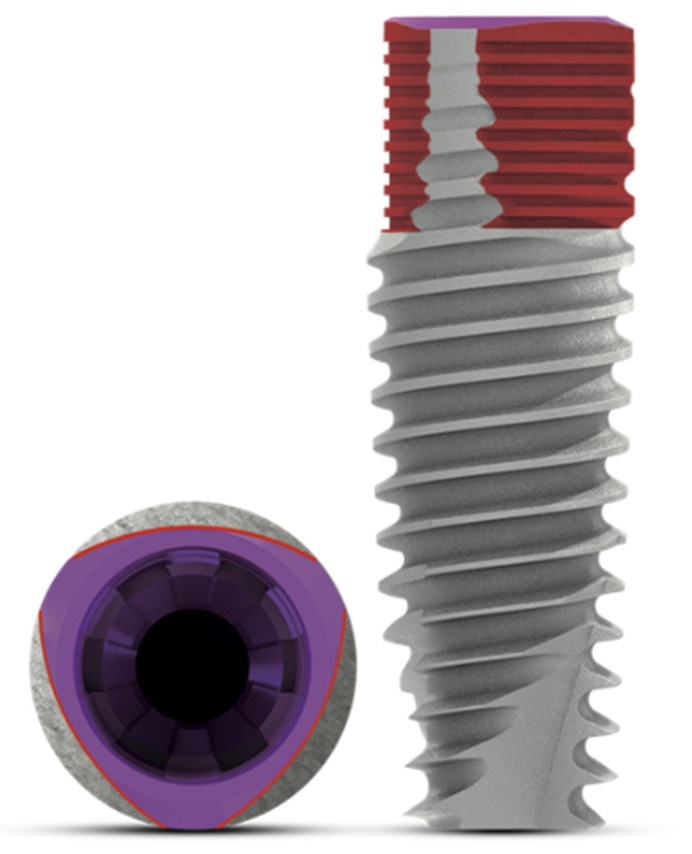
Conical connection platform and triangular shape of the coronal third of the V3 implant.

25 V3 (MIS Implants Technologies Ltd., Bar-Lev Industrial Park, Israel) implants were placed in 16 patients in both the maxilla and mandible. This novel triangular neck features gaps of 0.1 and 0.3 mm depending on the implant diameter; it incorporates the platform-switching feature and a 12° conical connection (Figure 1). The implant surface is sandblasted and acid-etched; it is also osseo-conductive in soft bone (Kim et al.) [33].

**Figure 2:**
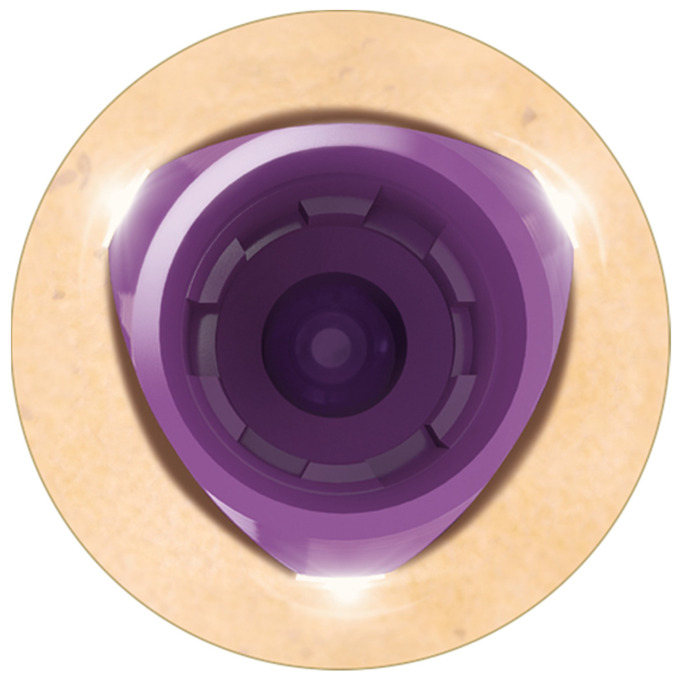
Occlusal view showing the flat area of the triangle oriented toward the buccal aspect of the ridge. The gap between the implant surface and surrounding bone can also be appreciated. Note the three areas of contact allowing primary stability.

The implants used were narrow platform 3.3 mm and standard 3.9 diameter implants with lengths ranging between 8- and 13-mm. Local anesthesia was administered using 4% articaine with 1:100,000 epinephrine (DFL, Rio de Janeiro, RJ, Brazil) then a full thickness flap was reflected using crestal incision. After performing the osteotomy in accordance with the recommendations of the manufacturer, the implants were placed in the healed bone ridge in such a way that the flat area was oriented toward the labial aspect of the ridge extending the implant away from the buccal crestal bone, as illustrated in (Figure 2). Insertion torque was ≥30 Ncm and sub-crestal placement did not exceed 0.5 mm on the buccal side; no regenerative procedure was performed. The flap was sutured using 5-0 monofilament nylon suture (Ethicon, Johnson & Johnson, New Brunswick, NJ, USA). A transgingival healing was allowed. Postoperative instructions included 25 mg of dexketoprofen for 2 days and local irrigation of chlorhexidine 0.12%, three times a day for 7 days. The patient returned one week after surgery for suture removal and postoperative assessment. Minimal pain and edema on the surgical site were observed with no signs of infection or adverse reactions. All patients receive a post-operative oral hygiene instruction, supra- and subgingival debridement using ultrasonic and hand instruments in the dentition after 1, 4 and 12 weeks of surgery.

### 2.3. Prosthetic Procedures

**Figure 3:**
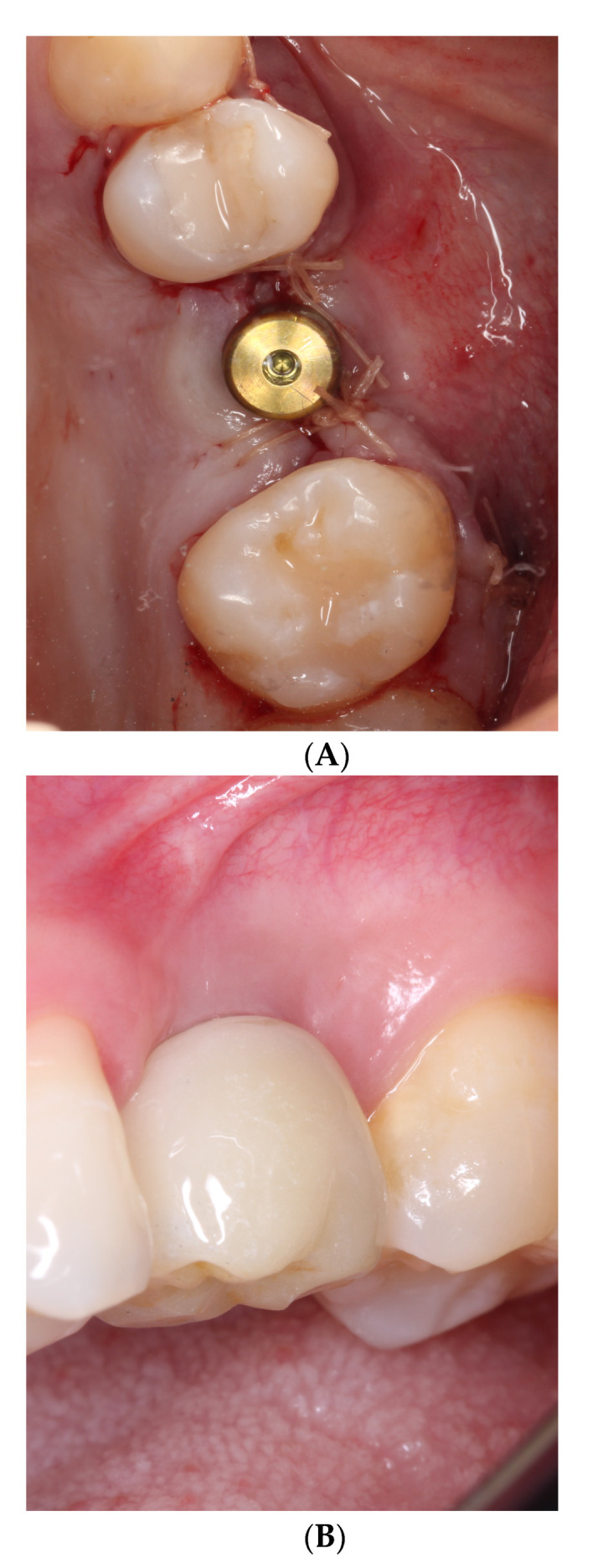
(**A**): Implant placement at the end of surgery. (**B**): Clinical follow-up at 2 years.

Implants were uncovered between 3 and 7 months through a slightly lingual crestal incision in order to preserve the greatest amount of keratinized tissue. After the healing period, a temporary cylinder was screwed to the implant and a temporary crown was cemented (3M Tempory Cement, Flemington, NJ, USA) and left for three weeks to gradually create a customized emergence profile. Final impressions were done using impression copings to register the position of the implant. An implant analog was placed and a silicone gingiva (Gingifast CAD Zhermack, Badia Polesine (RO), Italy) was placed around the analog at the interface of the emergence profile. Abutments were selected according to the requirements of each case, scanned and then the crowns were designed in the virtual model (Amman-Girrback Ceramill Map 400, Koblach, Austria). Conventional Zolid^®^ zirconia (Amman Girrbach, Koblach, Austria) was used for all crowns (screw-cemented retained crowns) and a five-axis ceramill motion 2 milling machines (Amman-Girrbach, Koblach, Austria) with a vestibular cutback performed the milling. Subsequently, the crowns were laminated and sintered in the oven (Ceramill Term 3 -Amman-Girrbach, Nowak Dental Supplies, Inc., Carriere, MS, USA) for 7 h. The final crowns were stratified with vintage ZR dentin and enamel ceramic (Shofu Inc. Dental Asia-Pacific Pte. Ltd., Singapore) on the buccal side and placed in the oven (Porgramat 5000 Ivoclar Vivadent, Amherst, NY, USA) at temperatures of 900 degrees Celsius. Eighteen crowns were screw-retained, whereby the access was sealed with Ketac Fil and flowable composite. The other 7 crowns were cement-retained, and the abutment was torqued at 30–35 Ncm, verifying its position with a radiograph and the crown finally cemented using resin-reinforced glass ionomer cement (GC Fuji Plus, Tokyo, Japan) (Figure 3A,B). The patients received oral hygiene instructions and full mouth professional mechanical plaque removal after 6, 12 and 18 months after receiving the final implant restoration.

### 2.4. Outcomes of the Study

The primary outcome of the present study was peri-implant crestal bone loss; it was measured immediately after delivery of the final implant-supported prosthesis (baseline) and at the final follow-up appointment with standardized periapical radiographs. The secondary outcomes were the clinical parameters measured at the mesial and distal aspect of each implant between baseline and final follow-up; they included peri-implant probing depth (PPD) and bleeding on probing (BoP).

### 2.5. Radiographic Examinations

**Figure 4:**
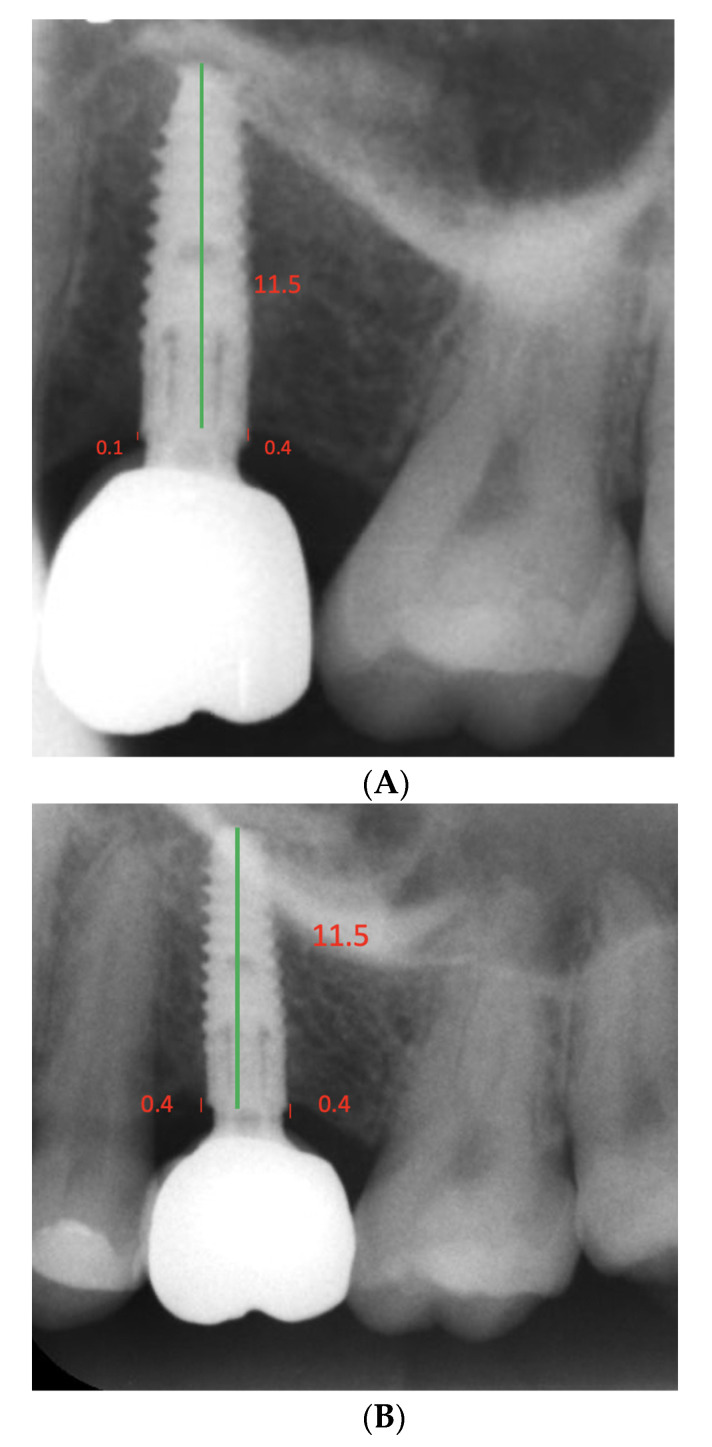
(**A**): Measurements of the hard tissue parameters using standardized long cone radiographs implant length (green vertical line). (**B**): Distance between the neck and the first bone-implant contact (red vertical line).

Periapical radiographs were taken and processed using a PSPiX^®^ imaging scanner (ACTEON^®^ group, Mérignac, France). The radiographs were taken the day that the definitive implant-supported restoration was delivered, and at the last postoperative recall. Radiographic measurements were performed using the Planmeca Romexis version 5.0 software (Planmeca Oy, Helsinki, Finland); comparisons between baseline and follow-up were carried out by a certified and calibrated radiologist using the related software (Romexis, Planmeca Oy, Helsinki, Finland). To assess peri-implant bone loss the implant length was measured; subsequently, the proximal bone height levels measured were measured mesially and distally from the implant shoulder to the first bone-implant contact (Figure 4A,B).

### 2.6. Clinical Measurements and Examinations

A single trained examiner performed all clinical recordings using a periodontal probe (PCPUNC-12; Hu-Friedy, Chicago, IL, USA). At the time as the final restoration was placed, the baseline clinical examination was taken and included recordings of:

(1) Peri-implant Probing depth (PPD): was measured to nearest 0.5 mm at the mesial and distal site.

(2) Bleeding on probing (BoP): A dichotomous score was given (0 = no bleeding; and 1 = bleeding) at six sites per implant, including mesial, medium, and distal sites of both buccal and palatal). The recordings of PPD and BOP were repeated for each participant at the final recall appointment.

### 2.7. Statistical Analysis

Peri-implant probing depth average of mesial, medium, and distal sites of both buccal and palatal (PPDMsB, PPDMB, PPDDB, PPDMsP, PPDMP, PPDDP) were analyzed with the paired *t*-test to compare the average probing depth between baseline and follow-up. BoP was converted to 0 (−) and 1 (+) and the average of six sites were calculated and compared using the paired *t*-test. For clinical attachment level, paired *t*-test was used to compare baseline and follow-up measurements. For radiographic evaluation, the paired *t*-test was used to examine the difference between the mesial and distal values, and location of the implant (maxilla vs. mandible).

## 3. Results

A total of 16 participants were enrolled in this study following the protocol described above. The proportion of men was (N = 4, 25%) and women (N = 12, 75%) with ages ranged from 28 to 65 years. The majority of the implants (N = 18) had a standard diameter platform, and the remaining seven implants were narrow platform. The most frequently used diameter was 3.9 mm, followed by 3.3 mm; implant lengths varied between 8 and 13 mm; only one narrow platform implant was 16 mm long. Twenty-five implants were placed with a distribution of 11 implants in the maxilla and 14 in the mandible. With regard to the position of the restorations, three were anterior, fifteen premolars, and eight were molars. Most of the implant restorations (18) were screwed, and seven were cemented. Eleven patients received single crowns, three patients received two crowns each, one patient thre3 and one patient five crowns. All implants achieved osseointegration and no biological or prosthetic complications were reported at any time during the healing period of the implants or the follow-up period. The minimum period of clinical and radiographic follow-up after the final restoration was delivered was of 15.6 months (range: 8 to 24 months) (Table 1).

Comparisons of radiographic measurements between baseline and follow-ups are shown in Table 2. With respect to the mean bone levels, at baseline the mesial and distal sites were 0.45 mm (0.47) and 0.57 mm (0.69), respectively. At follow-up, the measured bone levels were 0.59 mm (0.42) and 0.78 mm (0.59), respectively. No significant difference was observed between baseline and follow-up at the mesial (*p* = 0.30) or distal (*p* = 0.17) locations. Statistical significance was found between the baseline mesial and follow-up mesial sites in the maxilla (baseline: 0.39 (0.31); follow-up: 0.65 (0.30), *p* = 0.046), no other statistically significant differences were found between baseline and follow-up at the mesial or distal locations (Table 3).

The mean peri-implant pocket depth at follow-up was significantly higher than baseline for the maxillary implants (baseline: 1.70 ± 0.7 vs. follow-up: 2.20 ± 0.59, *p* = 0.0016); no statistical significance was observed among mandibular implants (Table 4).

## 4. Discussion

There are several studies that confirm that the implant neck features play an important role in implant (primary and secondary) stability and in the initial biologic process that leads to the formation of peri-implant tissues [34,35,36]. While implants with narrow and converging necks create less stress and pressure to the crestal bone of the alveolar ridge, implants with divergent necks can further compress the crestal bone, causing greater stress and future loss of peri-implant tissues [37,38]. In the present investigation, all implants were bone level implants. They achieved an initial high insertion torque and were placed with the flat portion of the implant neck facing the buccal bone; this resulted in areas of less bone compression and stress. In previous studies, it has been demonstrated that bone level implants with a rough surface in their coronal portion have a greater bone-to-implant contact when compared to polished necks [39,40]. It has been shown that implants with a polished neck are more recommended in patients with a history of periodontal disease or at risk of suffering of peri-implantitis such as patients with poor oral hygiene, smokers or systemically compromised individuals [36,38,41]. In the present study, clinical and radiographic results have been presented after a mean follow-up of 15.6 months. No significant difference between the radiographic baseline measurements and follow-up was observed, apart from in the mesial site of the maxilla (baseline: 0.39 ± 0.31; follow-up: 0.65 ± 0.30, *p* = 0.046). In a recent study, Li Manni et al. [29], conducted the first randomized controlled trial to compare the peri-implant bone loss and other clinical outcomes between the traditional and the triangular neck shape implants. The mean ±SD peri-implant interproximal bone loss 1 year after loading was 0.22 ± 0.30 mm for the triangular and 0.42 ± 0.67 mm for the circular implants necks (*p* = 0.25); our results are in line with their data. Noteworthy, these authors did not find any difference between the clinical and radiographic results of the triangular and circular neck implants in the posterior maxilla.

In the present study, no statistically significant differences were found for the clinical parameters between baseline and the follow-up. D’Avenia et al. [31]. conducted a study to evaluate the clinical and radiographic outcomes of the triangular neck implant design. The authors reported satisfactory esthetic, clinical and radiographic results after one year of function. In another study, Nevins et al. [32], recently conducted a human histological study using this specific implant design. Four patients received several implants for full-mouth reconstruction; in addition, eight triangular neck implants were placed in the healed edentulous maxillary or mandibular ridges. By the end of the final osteoctomy a 0.2 mm gap was allowed between the triangular implant neck and the surrounding bone, and the study implants were harvested after 6 months of submerged healing. The mean bone-implant contact (BIC) measured all over the implant surface was 68.58 ± 3.76% and no statistically significant differences between the BIC measured on the µCT and the histological sections were observed. The research mentioned above is the only human evidence to date providing histological results with this dental implant design.

The macro-design of an implant usually refers to the shape of the implant threads, body, and neck design. Additionally, this term includes the micro-morphology produced by the surface treatment with respect to the depth, size, and width of the roughness [42]. Degidi et al. [43], in a retrospective human study, evaluated histologically and histomorphometrically the bone response around 10 implants with a parallel-wall configuration, condensing thread macro-design, and self-tapping apex. High bone-implant contact percentages were found and the authors concluded that both the macrostructure and the microstructure participated to the high long-term survival and success of the implant. In another study, de Andrade et al. [44] evaluated the influence of implant macro-design when using different types of collar and thread designs on stress/strain distributions in a maxillary bone site. They showed that the collar design was the main factor affecting the stresses/strains at the cortical bone level. Recently, Montemezzi et al. [45]. investigated whether a different implant neck design (rough wide-neck implants vs. rough reduced-neck implants) could affect survival rate and peri-implant tissue health in a cohort of 97 disease-free partially edentulous patients. After two-year follow-up the survival rates were similar (96.61% vs. 95.82%). The V3 implants present in its design a roughness surface and micro-morphology because of a sandblasting and acid-etching treatment. In addition, it presents micro-rings on the neck of the implant that have been shown to facilitate an increase in bone-to-implant-contacts, thus reducing the loss of peri-implant marginal bone [30,31,32]. Furthermore, the flat sides leaving a small gap have been shown to speed-up the process of bone formation when compared to the part of the implant in full contact with the cortical bone [46].

Different in vivo studies [27,30] have been conducted to evaluate the unique triangular shape of the implant neck and conclude that similar percentage of osseointegration and greater buccal thickness of peri-implant hard tissue may be expected in this implant, even though conventional implant design seems to obtain more height values of the crestal bone compared with V3 implants, although the differences were not statistically significant. On the other hand, the stability of the V3 implant has recently been studied in a randomized prospective longitudinal clinical study, demonstrating similar results with round neck implants in secondary stability values after 6 weeks of healing [28].

In the present study, the implants were placed in healed alveolar ridges with different width measurements, without the need for grafting procedures. It can be hypothesized that placing the flat portion of the triangle toward the buccal bone may allow greater distance from that critical area, reducing the risk of bone loss due to compression, stress, and remodeling, significantly improving the stability for hard and soft peri-implant tissues. Therefore, it is the authors’ opinion that this implant could be used in cases of narrower alveolar ridges and cases with greater esthetic demands.

The implant used in the present study uses a platform-switching design concept with a 12° conical connection that may lead to peri-implant tissue preservation, through an enhancement the horizontal thickness of soft tissue, hermetic seal between abutment-implant interface and reducing micro-movements. Several studies have investigated the concept of platform switching in the preservation of peri-implant tissues, where the abutment is narrower than the implant diameter. In general, the results showed that the platform switch concept preserve, maintain and in some cases increase the soft and hard tissues around the implants [14,47,48,49].

Previous and recent studies with immediate implant placement have shown that intentionally placing the implant slightly palatally could allow the formation of a gap between the implant surface and the buccal bone ridge, which can be occupied by the clot or filled with some regeneration biomaterial. This may lead to a better emergence profile of the restoration and an adequate amount of peri-implant tissue in the long-term. Although the present investigation only included partially edentulous patients with healed alveolar ridges, the V3 implant is especially useful in cases of immediate implant placement, because its triangular design in the coronal third allows three contact points that facilitate primary mechanical stability, and creating spaces between the implant surface and adjacent bone, that facilitate formation and stabilization of the blood clot and bone regeneration.

There are some limitations in the present study that should be mentioned: population size, limited number of implants and patients, upper and lower jaws both treated, mixed cemented and screw-retained restorations. Another possible limitation was that a standardized method of taking periapical radiographs was not used. Nor was a control group used to be able to compare these results. Finally, in the present study, the follow-up periods was limited.

## 5. Conclusions

Within the limitations of this prospective clinical study, the present results showed that this unique implant design represented a predictable solution for the rehabilitation of patients requiring dental implants after a mean follow-up of 15.6 months and could be used by the clinician in a safe and effective way in partially edentulous patients. More clinical investigations in partially and totally edentulous patients using immediate or delayed placement protocols are needed to assess the mechanical and biological behavior of the soft and hard tissues around triangular implant design.

## Figures and Tables

**Table 1:** Demographic data and dimensions of implants.

				**N (%)**		
Female				12 (75)		
Male				4 (25)		
**Patient number and number of implants**						
**Patient Number**				**Number of Implants**		
1				1		
2				1		
3				1		
4				2		
5				1		
6				1		
7				5		
8				2		
9				1		
10				1		
11				2		
12				1		
13				3		
14				1		
15				1		
16				1		
**Age 45.82 (13.15) years**						
**Length and diameter**						
**Diameter**				**N (%)**		
3.3				7 (28)		
3.9				18 (72)		
**Length**				**N (%)**		
8				4 (16)		
10				8 (32)		
11.5				6 (24)		
13				6 (24)		
16				1 (4)		
**Patient implant details**						
**Patient Number**	**Age**	**Sex**	**Implant Tooth**	**Location**	**Diameter Length**	**Follow-Up Length (Mo.)**
1	35	F	13	Maxilla	3.90 × 11.5	12
2	60	F	4	Maxilla	3.90 × 10	12
3	50	F	20	Mandible	3.90 × 11.5	8
4	57	F	3	Maxilla	3.30 × 10	12
4	57	F	4	Maxilla	3.90 × 8	12
5	38	F	19	Mandible	3.90 × 10	17
6	57	F	5	Maxilla	3.30 × 10	9
7	65	F	18	Mandible	3.90 × 8	24
7	65	F	17	Mandible	3.90 × 10	24
7	65	F	28	Mandible	3.90 × 11.5	13
7	65	F	29	Mandible	3.90 × 11.5	13
7	65	F	30	Mandible	3.90 × 10	13
8	65	F	27	Mandible	3.90 × 13	11
8	65	F	28	Mandible	3.90 × 13	11
9	33	M	29	Mandible	3.30 × 10	16
10	55	F	4	Maxilla	3.30 × 11.5	15
11	50	F	5	Maxilla	3.30 × 13	21
11	50	F	4	Maxilla	3.30 × 13	21
12	32	M	5	Maxilla	3.90 × 13	16
13	45	F	18	Mandible	3.90 × 8	24
13	45	F	19	Mandible	3.90 × 10	24
13	45	F	30	Mandible	3.90 × 8	24
14	35	F	28	Mandible	3.90 × 13	20
15	28	M	8	Maxilla	3.30 × 16	12
16	28	M	9	Maxilla	3.90 × 11.5	16
						Mean follow up 15.6 months

**Table 2:** Mean bone levels.

Bone Levels	Baseline Mean (SD)	Follow-Up Mean (SD)	*p*-Value
Mesial	0.45 (0.47)	0.59 (0.42)	0.30
Distal	0.57 (0.69)	0.78 (0.59)	0.17

**Table 3:** Bone levels according to site location.

		Baseline Mean (SD)	Follow-Up Mean (SD)	*p*-Value
Maxilla (N = 11)	Mesial	0.39 (0.31)	0.65 (0.30)	0.046
	Distal	0.44 (0.29)	0.78 (0.39)	0.09
Mandible (N = 14)	Mesial	0.5 (0.57)	0.54 (0.50)	0.87
	Distal	0.68 (0.88)	0.79 (0.73)	0.65

**Table 4:** Mean peri-implant pocket depth of maxilla and mandible sites at baseline and follow-up.

	Baseline		Follow-Up		
	N	Mean (SD)	N	Mean (SD)	*p*-Value
Maxilla PPD	11	1.70 (0.37)	11	2.20 (0.59)	0.006
Mandible PPD	14	1.46 (0.35)	14	1.57 (0.60)	0.60

## Data Availability

Not applicable.
